# Brain activation in frontotemporal and Alzheimer’s dementia: a functional near-infrared spectroscopy study

**DOI:** 10.1186/s13195-016-0224-8

**Published:** 2016-12-08

**Authors:** Florian G. Metzger, Betti Schopp, Florian B. Haeussinger, Katja Dehnen, Matthis Synofzik, Andreas J. Fallgatter, Ann-Christine Ehlis

**Affiliations:** 1 Department of Psychiatry and Psychotherapy, University Hospital of Tuebingen, Calwerstraße 14, 72076 Tuebingen, Germany; 2 Geriatric Center at the University Hospital of Tuebingen, Calwerstraße 14, 72076 Tuebingen, Germany; 3 Center of Neurology, Department of Neurodegeneration and Hertie Institute for Clinical Brain Research, University Hospital of Tuebingen, Hoppe-Seyler-Straße 3, 72076 Tuebingen, Germany; 4 German Center of Neurodegenerative Disorders (DZNE), University Hospital of Tuebingen, Otfried-Müller-Straße 23, 72076 Tuebingen, Germany

**Keywords:** Functional near-infrared spectroscopy, Alzheimer’s dementia, Frontotemporal dementia

## Abstract

**Background:**

Frontotemporal dementia is an increasingly studied disease, the underlying functional impairments on a neurobiological level of which have not been fully understood. Patients with the behavioral-subtype frontotemporal dementia (bvFTD) are particularly challenging for clinical measurements such as functional imaging due to their behavioral symptoms. Here, an alternative imaging method, functional near-infrared spectroscopy (fNIRS), is introduced to measure task-related cortical brain activation based on blood oxygenation. The current study investigated differences in cortical activation patterns of patients with bvFTD, Alzheimer’s dementia (AD), and healthy elderly subjects measured by fNIRS.

**Method:**

Eight probable bvFTD patients completed the semantic, phonological, and control conditions of a verbal fluency task. Eight AD patients and eight healthy controls were compared on the same task. Simultaneously, an fNIRS measurement was conducted and analyzed using a correction method based on the expected negative correlation between oxygenated and deoxygenated hemoglobin.

**Results:**

Healthy controls show an increase in cortical activation measured in frontoparietal areas such as the dorsolateral prefrontal cortex. The activation pattern of patients with AD is similar, but weaker. In contrast, bvFTD patients show a more frontopolar pattern, with activation of Broca’s area, instead of the dorsolateral prefrontal cortex and the superior temporal gyrus. The frontoparietal compensation mechanisms, seen in the healthy elderly, were missing in bvFTD patients.

**Conclusion:**

Different frontoparietal cortical activation patterns may indicate a correlate of diverse pathophysiological mechanisms of AD and bvFTD during verbal fluency processing. The AD pattern is weaker and more similar to the healthy pattern, whereas the bvFTD pattern is qualitatively different, namely more frontopolar and without frontoparietal compensation activation. It adheres to a change of cortical activation during the course of the disease.

**Electronic supplementary material:**

The online version of this article (doi:10.1186/s13195-016-0224-8) contains supplementary material, which is available to authorized users.

## Background

Frontotemporal lobar degeneration is the second most common neurodegenerative disease causing dementia besides Alzheimer’s dementia (AD). Within frontotemporal lobar degeneration, the behavioral variant of the frontotemporal dementia (bvFTD) is the most common subtype [1]. While dementia in AD is characterized by impairments in working memory and episodic memory [2], in bvFTD the main symptoms are social disinhibition, deficits in motivation, striking changes in behavior, and executive dysfunction [1, 3, 4]. Despite these characteristics, cognitive symptoms in both entities may overlap. Memory might be less impaired than in AD, but episodic memory can be disturbed in even early stages of bvFTD, which may result in misdiagnoses of AD [5]. This overlap and heterogeneity increases the difficulty of differential diagnosis between AD and bvFTD, especially in early stages of the disease. To improve the diagnostic accuracy, the development of biomarkers and a better understanding of the underlying cortical mechanisms would be very helpful.

The genetic, molecular, and neuropathological underpinnings of bvFTD have been increasingly unraveled in the last years [6, 7]. Genetic and molecular profiles with the most common variants of nucleinacid-binding proteins such as FUS, TDP-43, C9orf72, or Progranulin contribute only partly to diagnose bvFTD patients [8]. Neuroimaging is another neurobiological method used for differentiation and, in particular, also for deeper insights into underlying functional brain networks. In bvFTD, structural imaging presents similarly to AD: an atrophy in the anterior cingulate, anterior insula, and subcortical structures, particularly in the frontal and temporal lobes [9], possibly characterizing different subtypes [10]. A further developed structural method is the structural connectivity analysis (diffusion tensor imaging) which is used for differential diagnosis by measuring fiber tracts. As expected, the focus of abnormalities in bvFTD are frontal and temporal regions but with more and more posterior damage during the course of the disease [11, 12]. Recently, in contrast to AD, functional imaging studies with bvFTD patients focusing on functional connectivity measurements have increasingly elucidated disease-specific network changes. The main focus of the last decade has been on the default-mode network, with a posterior connectivity of hippocampus, cingulum, temporal, and parietal gyri and the salience network with a more anterior connectivity of cingulum, frontoinsular, and orbitofrontal gyri. While the default-mode network was found to be abnormal in AD and somewhat less impaired in bvFTD [13, 14], the salience network is characteristically changed in bvFTD [15, 16] with only small changes in AD. In direct comparisons, the salience network seems to differentiate more precisely between both neurodegenerative diseases [17]. While this has been a common procedure in AD, initial results from studies combining biomarkers and imaging in bvFTD show a significant influence of C9orf72 or granulin genotypes on network connectivity as measured with functional magnetic resonance imaging (fMRI) [18, 19].

Functional imaging studies other than resting-state measurements are quite rare in bvFTD, and only a few studies have been conducted [20, 21]. The very small number of task-related studies using fMRI is possibly due to the characteristics of fMRI, although fMRI is the most frequently applied non-invasive neuroimaging method. In more detail, fMRI is uncomfortable, particularly for cognitively impaired, behaviorally challenging patients such as those with bvFTD. The scanner is noisy and narrow, and avoiding movement artifacts during the measurements is crucial, therefore requiring an absolutely still resting position from the participants. The tasks must be performed in an unnatural setting devoid of face-to-face contact, which is otherwise the norm during neuropsychological testing. Spoken or written answers are very difficult to record. Regarding the incremental abnormal behavior in progressing bvFTD, the unnatural setting is particularly significant. This may account for the lack of task-related studies using fMRI. In contrast to fMRI, a functional near-infrared spectroscopy (fNIRS) measurement is closer to the setting of a neuropsychological test situation: the participant is sitting on a chair, has close contact to the interviewer, and verbal answers are allowed and required. The basic principles of fNIRS and fMRI are the same; both methods assess the blood oxygenation level-dependent (BOLD) response. Near-infrared light penetrates biological tissue including skin, muscles, and skullcap. Oxygenated and deoxygenated hemoglobin absorb the near-infrared light with a focus on the event-related changes in the small vessels. In contrast to fMRI, a higher temporal resolution is possible (sampling rate is 10 Hz). fNIRS is very suitable for investigating paradigms associated with cognition [22], but also functional connectivity using resting-state measurements [23]. Additionally, fNIRS is a suitable and well accepted method for elderly and/or cognitively impaired subjects.

Many different cognitive tasks have been used in fNIRS studies in elderly subjects so far. The Benton Line orientation task assesses visuoconstruction and clearly differentiates AD patients and controls [24]. The Trail Making test is a common test used to evaluate executive functions, and requires only slight modification to the paper-and-pencil version in the fNIRS setting [23, 25]. The Verbal Fluency Task (VFT) investigates language abilities as well as executive functions, and is therefore often used in different neuropsychological test batteries examining cognitive functions in the elderly. The VFT is based on retrieval of nouns with commonalities in two different areas. In the semantic version, the subject has to produce as many nouns of a specific category (e.g., animals or flowers) as possible. In the phonological task, as many nouns as possible beginning with a certain letter have to be found. The VFT is also the most intensively examined paradigm combined with fNIRS (in the elderly) showing a decreasing activation in prefrontal areas [26, 27] with increasing age. In both conditions, bilateral hemodynamic responses (pronounced in the left hemisphere) within inferior and middle frontal areas have been measured by using multichannel fNIRS in younger as well as in elderly healthy controls [26, 28, 29]. Participants usually perceive the phonological condition as more difficult, so the activation effect of the phonological condition is correspondingly stronger than the effect of the semantic condition [28]. The activation effect is apparent for the cognitive impairment in AD patients in contrast to healthy controls [30, 31]. Even for the assessment of medication effects (e.g., cholinesterase inhibitors), the VFT proved to be suitable [30, 32].

In the present study, the VFT paradigm was used during an fNIRS recording to compare bvFTD patients to a clinical control group of AD patients as well as age-matched healthy control participants. In bvFTD, task-related imaging has rarely been conducted because of behavioral changes and associated difficulties for the application of imaging methods such as fMRI. Previous findings on differences between frontotemporal dementia and AD concerning resting-state activation lead to the question regarding possibly different cortical activation patterns in a task-related design.

## Methods

### Subjects

Eight subjects (three female and five male) aged between 60 and 79 years with the probable (*n* = 6) or definitive (*n* = 2) diagnosis of bvFTD according to the consensus criteria [4] were included. Eight patients (prior to medication) with the diagnosis of probable AD according to the criteria of McKhann [2] were added as a clinical comparison group and matched concerning age, gender, education, and behavioral data in the VFT to ensure optimal comparability. Additionally, eight gender-, age-, education- and medication-matched (to the bvFTD group; propensity score matching) healthy elderly control participants (HC) were screened to exclude memory complaints, drug or alcohol abuse, major psychiatric disorders, and neurological or cerebrovascular diseases from the TREND study sample (Tuebinger evaluation of Risk factors for Early detection of NeuroDegeneration). The healthy sample received the fNIRS measurement and the neuropsychological assessment in the course of the TREND study. All were examined with an assessment consisting of medical history, physical examination, and extensive neuropsychological tests. The bvFTD and AD patients were in- or outpatients of the University Hospital of Psychiatry and Psychotherapy of Tuebingen (Germany). All participants underwent a CERAD-Plus (Consortium to Establish a Registry for Alzheimer’s Disease) test battery, consisting of neuropsychological tests examining different cognitive domains [33] such as semantic and phonemic fluency (similar to the VFT), word retrieval (short version of the Boston Naming Test), constructional praxis and visual memory (using four figures to copy and free recall), verbal memory (using three learning and immediate recall trials of a 10-word list, delayed recall and discrimination), executive function (Trail Making Test B), and motor speed (Trail Making Test A).

### fNIRS

**Fig. 1:**
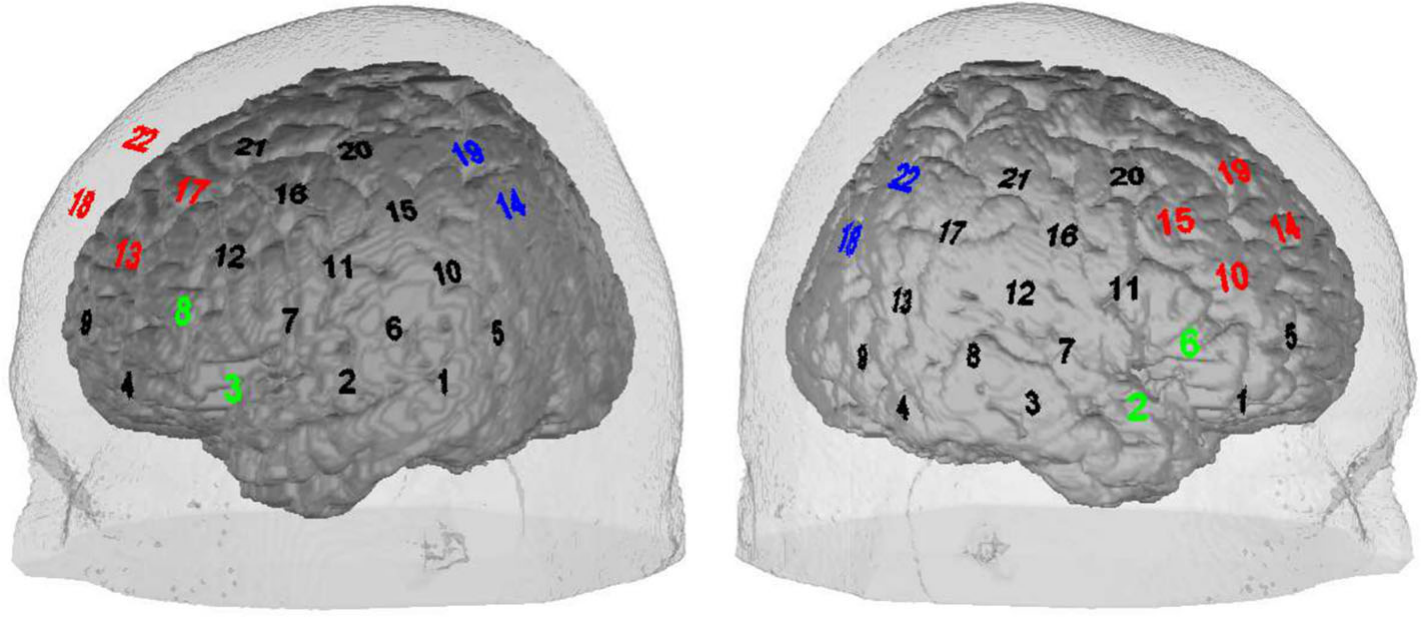
Localization of the NIRS probeset over the left and the right frontotemporal cortex. The *green* numbers indicates the region of interest (ROI) over Broca’s area, the *red* numbers the dorsolateral prefrontal ROI, and the *blue* numbers the parietal ROI

To measure cortical activation, fNIRS was used while performing the Verbal Fluency Task (VFT). A multi-channel NIRS system (ETG-4000 Optical Topography System; Hitachi Medical Co., Japan) with a temporal resolution of 10 Hz was used for all measurements. Near-infrared light penetrates biological tissue including skin, bone, and cerebrospinal fluid, and is mostly absorbed by hemoglobin with different wavelengths (695 nm ± 20 nm and 830 nm ± 20 nm) for oxygenated (O_2_Hb) and deoxygenated hemoglobin (HHb) [34, 35]. Therefore, the measurement of the cortical change of O_2_Hb and HHb is possible. The near-infrared light is emitted by one type of optode, the emitters, and absorbed by another, the detectors, with a fixed inter-optode distance of 30 mm in the system used. In this study, two 3 × 5 optode probesets, resulting in 22 channels each, were used which were fastened with elastic straps on the head of each subject. Channel 2 of the left probeset was positioned over T3 and the corresponding channel 3 of the right probeset over T4, following the international 10–20 system [36]. Large parts of the prefrontal and the temporal cortex of both hemispheres were covered by this orientation of NIRS optodes (see Fig. 1). For allocation of channels in relation to the Brodmann areas (BAs) according to the method of Singh et al. [37–39], see Table 1.

**Table 1:** Allocation of the Brodmann areas to the channel areas according to the method of Singh et al. [37–39]

Channels	Accuracy (%)	Brodmann areas	
*Left*			
9	56	10	Frontopolar area
10	61	2	Primary somatosensory cortex
15	36	3	Primary somatosensory cortex
11	71	43	Subcentral area
12	53	44	Pars opercularis, part of Broca’s area
1, 2	77, 47	21	Middle temporal gyrus
5, 6	92, 52	22	Superior temporal gyrus
14, 19	100, 75	40	Supramaginal gyrus, part of Wernicke’s area
3, 8	58, 1	45	Pars triangularis, part of Broca’s area
4, 13	99, 63	46	Dorsolateral prefrontal cortex
7, 16, 20	68, 78, 74	6	Pre-motor and supplementary motor cortex
17, 18, 21, 22	50, 56, 82, 82	9	Dorsolateral prefrontal cortex
*Right*			
21	47	1	Primary somatosensory cortex
12	48	2	Primary somatosensory cortex
4	49	20	Inferior temporal gyrus
3	85	21	Middle temporal gyrus
9	95	37	Fusiform gyrus
2	63	38	Temporopolar area
18	90	39	Angular gyrus, part of Wernicke’s area
16	31	43	Subcentral area
15	77	44	Pars opercularis, part of Broca’s area
19	97	9	Dorsolateral prefrontal cortex
17, 22	74, 99	40	Supramaginal gyrus, part of Wernicke’s area
6, 1	82, 1	45	Pars triangularis, part of Broca’s area
11, 2	67, 79	6	Pre-motor and supplementary motor cortex
7, 8, 13	32, 80, 78	22	Superior temporal gyrus
1, 5, 14	46, 92, 74	46	Dorsolateral prefrontal cortex

Accuracy refers to the accordance of the Brodmann areas to the position of the channels

### The Verbal Fluency Task (VFT)

All participants performed a VFT under three different conditions, which has been described before [26, 30]. Each condition lasted 30 s with pauses of 30 s between the different conditions; all three conditions were repeated three times. The first condition was the phonological part: the subjects were instructed to produce nouns starting with a specific letter (A, F, M, etc.) without quoting names. The second part of the task, the semantic part, was naming words of a specific category (professions, fruits, flowers, etc.). The content of both repeated conditions was pseudo-randomized for each follow-up measurement and did not overlap with the letters and categories used in the CERAD-Plus test battery. In the third condition (control condition), the subjects had to slowly produce the name of the weekdays, starting with Monday, until they were told to stop. Overall, the measurement took 9 min. Regarding previous fNIRS studies, an acceptable test-retest reliability has been shown at group level for this task [40]. Learning effects of the VFT were not assumed to occur, particularly for participants with dementia [41].

### Data and statistical analysis

For preprocessing of the concentration changes of O_2_Hb and HHb, the software of the fNIRS device was used. First, in order to exclude high frequency artifacts of the signal, a moving average was calculated using a time window of 5 s. Second, a linear fit for each block was conducted in order to exclude slow drifts in the NIRS signal. For this purpose, a 10-s baseline before the 30-s activation task and 20 s after the task were used as pre- and post-task baseline, respectively. The data of the three repetitions for each condition were averaged and exported.

The data were processed further and the image generation was realized using Matlab® R2009b (MathWorks Inc., Nattic, USA) and customized analysis routines. A correction method based on the expected negative correlation between oxygenated and deoxygenated hemoglobin dynamics (correlation-based signal improvement (CBSI)) was used in order to eliminate smaller artifacts related to, for example, body motion [42]. Afterwards, a correction using common average reference (CAR) was used to reduce arousal artifacts; channels were automatically screened for remaining artifacts based on a variance criterion and were automatically interpolated by surrounding channels (6% of the channels). Thereafter, a manual interpolation was conducted for channels with amplitudes exceeding ±0.5 mmol × mm/l. All interpolations with surrounding channels followed a Gaussian distribution, i.e., the closeness of a channel to an interpolated channel corresponds to the impact on interpolation. Then, the average during the interval spanning 10–30 s of the activation period of each task and each participant was calculated. Statistical analyses of these averages were performed using IBM SPSS Statistics 22 (SPSS Inc., an IBM Company).

For behavioral data analysis, VFT performance (number of correctly generated words) was averaged over the three repetitions, and each average of the three conditions (semantic vs. phonological fluency vs. control condition) was compared between the diagnostic groups (bvFTD vs. AD vs. HC) using a univariate analysis of variance (ANOVA).

Unspecific effects caused by articulation and speech movements on the NIRS data were controlled for by a subtraction of weekday activation from the two active fluency tasks (semantic and phonological fluency). In a first (exploratory) analysis, the fNIRS data of the two active conditions (semantic and phonological fluency task) were contrasted with the control task (weekdays) for each channel of both probesets and illustrated by maps of the effect sizes (Cohen’s *d*). Afterwards, group comparisons were performed using effect sizes to illustrate group differences in statistical activation maps due to the small sample size. As a further step of analysis, the brain activation in three regions of interest (ROI) was defined and correlated with behavioral data: Broca’s area is a target region for detecting classical VFT effects in younger healthy controls, and so we formed a ROI due to theoretical considerations and previous findings concerning VFT performance [43] (left probeset: channels #3 and #8; right: #2 and #6). Based on previous findings concerning the VFT in aged and cognitively impaired subjects, we defined two additional ROIs in the dorsolateral prefrontal cortex (DLPFC; left: #13, 17, 18, 22; right: #10, 14, 15, 19) and the parietal cortex (left: #14 and 19; right: #18 and 22) [26, 32]. (For an overview of the ROIs in relation to the probesets see Fig. 1.) Pearson’s correlation coefficient was calculated for CBSI amplitudes of the ROIs (mean of the single channels) and behavioral measures (produced words in each condition and each diagnostic group). To correct for multiple statistical testing, the Bonferroni-Holm correction for the number of ROIs was used here [44].

## Results

### Demographic and behavioral data

The HC group was age-matched to the bvFTD group and were significantly younger than the AD group (AD vs. HC: *t* = 3.11, df = 14, *p* = 0.008); the contrast between the bvFTD group and the HC group, and the bvFTD and AD groups did not reach statistical significance in a post-hoc *t* test. The three groups (bvFTD, AD, HC) differed significantly in all three conditions of the VFT; for details see Table 2 (one-way ANOVA). Using post-hoc *t* tests, the word production in the phonological condition was significantly lower in the AD as well as the bvFTD group as compared to healthy controls (AD vs. HC: *t* = –2.76, df = 14, *p* = 0.015; bvFTD vs. HC: *t* = –2.58, df = 14, *p* = 0.022); the same was true for the semantic condition (AD vs. HC: *t* = –5.15, df = 14, *p* < 0.001; bvFTD vs. HC: *t* = –3.05, df = 14, *p* = 0.009). Word production in the control (weekdays) condition was significantly lower in the healthy group than in both demented groups (AD vs. HC: *t* = 2.69, df = 14, *p* = 0.017; bvFTD vs. HC: *t* = 2.39, df = 11.5, *p* = 0.035). Apparently, the demented participants did not follow the instruction to pronounce the control condition in a speed comparable to the active task conditions as precisely as did the healthy controls.

**Table 2:** Demographics and behavioral data

	bvFTD	AD	HC	*p*
Age (years)	67.6 ± 9.8	74.3 ± 4.5	65.5 ± 6.5	0.064
Gender (female/male)	3/5	3/5	3/5	
Number of words in VFT				
Phonologic	3.4 ± 2.0	2.8 ± 2.5	5.7 ± 1.5	0.026
Semantic	6.3 ± 2.6	4.8 ± 2.0	9.7 ± 1.8	0.001
Weekdays	11.4 ± 2.0	11.1 ± 1.4	9.4 ± 1.2	0.039

*AD* Alzheimer’s dementia, *bvFTD* behavioral variant of frontotemporal dementia, *HC* healthy controls, *VFT* Verbal Fluency Task

### fNIRS data

Investigating the brain activation using fNIRS, the contrast of the active phases of the VFT and the control condition (weekdays) by means of relative changes in CBSI-corrected values was considered in a first step of the analysis. In the HC group, increased activation for the VFT “letter condition vs. weekdays” was found in the left DLPFC (BA 9 and 46, channels #13, 18, 22; all *d* > 0.8) and the left supplementary motor area (SMA; BA 6, channel #20; *d* = 0.95) as well as the right primary somatosensory cortex and Wernicke’s area (BA 1 and 40, channels #21 and 22; *d* > 0.9), whereas the left middle and superior temporal gyrus (MTG and STG; BA 21 and 22, channels #1, 5, 6; *d* > 0.9) and a part of Broca’s area and of the primary somatosensory cortex showed a decrease in activation (BA 2 and 45, channels #3 and 10; *d* > 0.9). In the contrast “category task vs. weekdays” a similar pattern was observed with activation in the DLPFC in both hemispheres (BA 9 and 46, channels #13, 17 left (*d* > 0.8) and #14, 19 right (*d* > 0.8)) and left Wernicke’s area (BA 40, channel #19; *d* > 0.9) and decreased activation in the left MTG and STG (BA 21 and 22, channels #2, 6, *d* > 1.0) and additionally left Broca’s, subcentral area/SMA (BA 6, 43, 45, channels #3, 7, 11, *d* > 1.0) and right MTG (BA 21, channel #3, *d* > 0.9).

The activation pattern of the AD group was, in both contrasts, quite similar to the HC but much weaker, particularly in the left hemisphere (“letter vs. weekdays”: no significant channel; “category task vs. weekdays”: MTG, Broca’s area, Wernicke’s area and DLPFC (BA 21, 45, 40, 9, channels #2, 3, 14, 18, 22; *d* > 0.8). In the right hemisphere, both contrasts were nearly the same as in the HC group with activation in the inferior temporal gyrus (ITG; BA 20, channel #4; *d* = 0.94) in the “letter vs. weekdays contrast”, and MTG (BA 3, channel #21; *d* = 0.90) and primary somatosensory cortex (BA 1, channel #21) in the “category task vs. weekdays” contrast.

In the bvFTD group, the activation pattern was characterized by an increased activation in left Broca’s area (BA 45, channel #8; *d* = 0.86) in both contrasts: the right temporopolar area and the STG (BA 38 and 22, channel #2 and 7; *d* > 0.8) in the letter contrast, and right Broca’s area (BA 45, channel #6; *d* = 1.08) in the category contrast. Decreased activation was observed in the left MTG and STG (BA 21 and 22, channels #1, 5 in the category contrast; *d* > 0.9) and the right STG and Wernicke’s area (BA 22 and 39, channels #13 and 18; *d* > 0.8) in both contrasts.

**Fig. 2:**
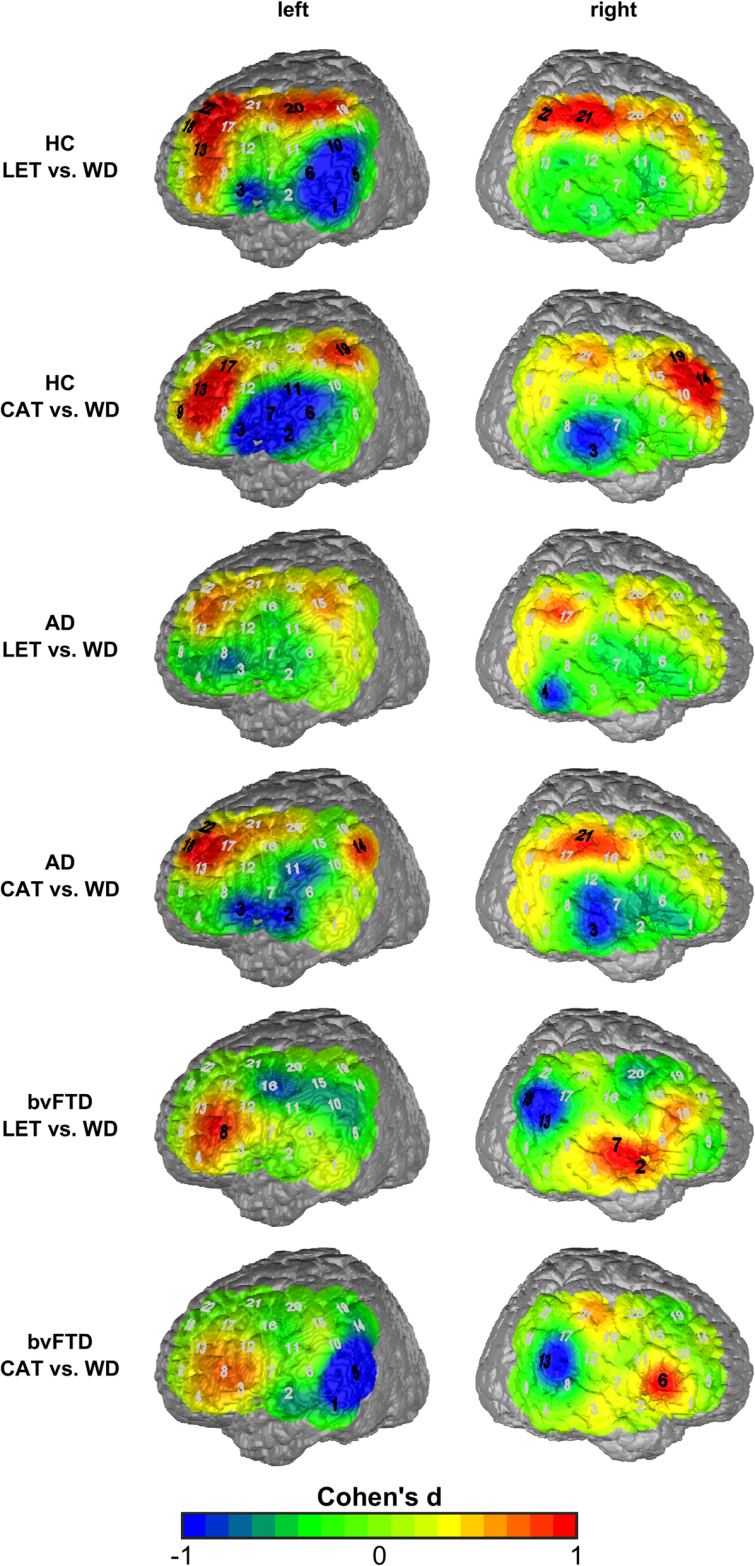
Maps of the effects sizes contrasting the letter condition, the category condition, and the weekday condition in the three groups (*black bold* signifies significant channels). *AD* Alzheimer’s dementia, *bvFTD* behavioral variant of frontotemporal dementia, *CAT* category (semantic) condition, *HC* healthy controls, *LET* letter (phonematic) condition, *WD* weekday (control) condition

The effect size maps of the mentioned contrasts, separated by diagnostic group, are shown in Fig. 2 (for detailed statistical data see Additional file 1). The channels mentioned in the text are marked by black bold in the figures, corresponding to an (uncorrected) significance in the *t* test.

**Fig. 3:**
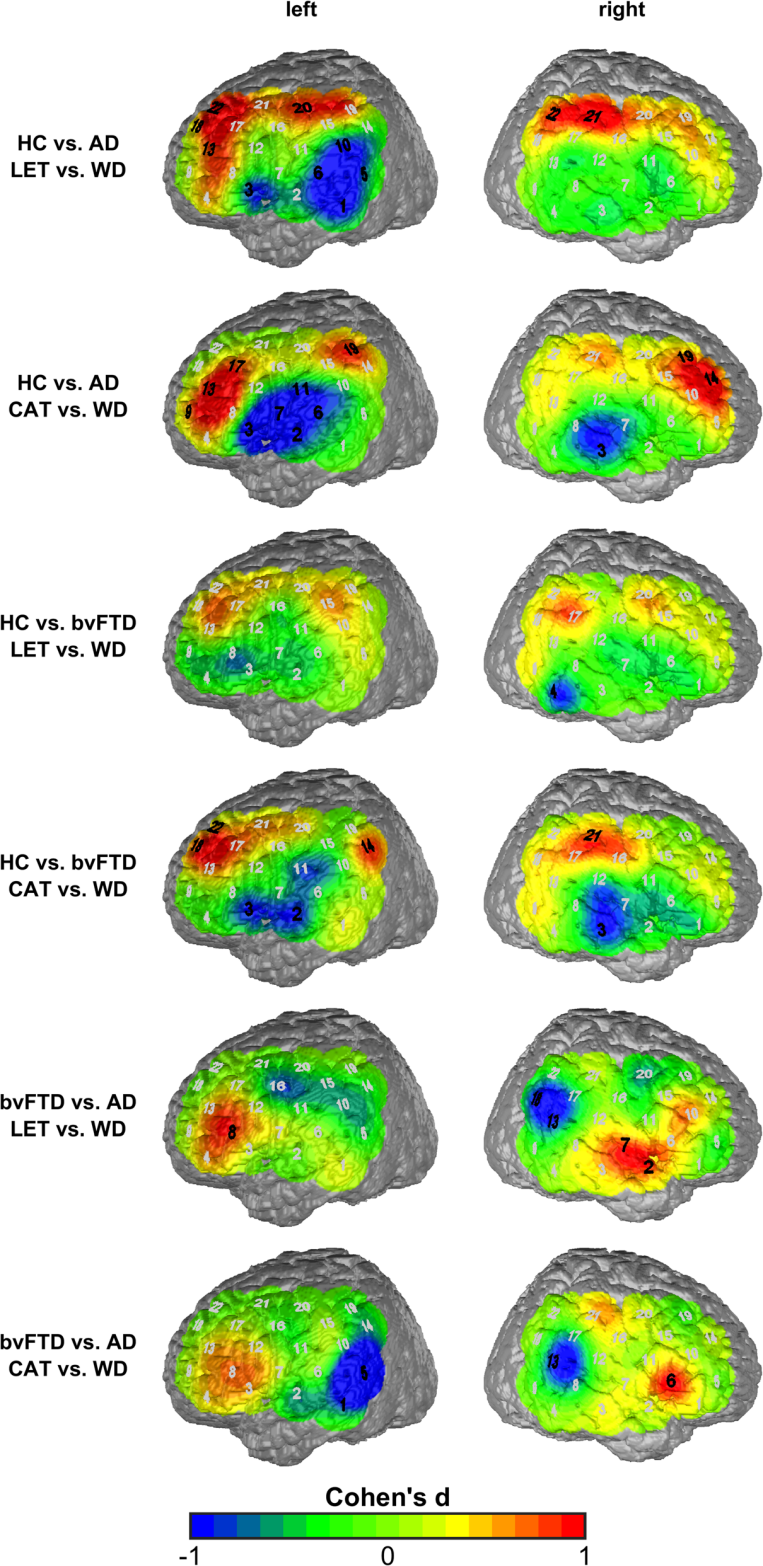
Maps of the effects sizes contrasting two groups each (*black bold* signifies significant channels). *AD* Alzheimer’s dementia, *bvFTD* behavioral variant of frontotemporal dementia, *CAT* category (semantic) condition, *HC* healthy controls, *LET* letter (phonematic) condition, *WD* weekday (control) condition

For the second step of the analysis, diagnostic groups were directly compared (Fig. 3; the channels mentioned in the text correspond to (statistically significant) channels marked in black; for detailed statistical data see Additional file 2). Here, the contrast of the groups HC vs. AD showed higher activation (in HC) in the letter contrast with a focus on left Broca’s area and DLPFC (BA 45 and 46), and lower activation in the area between left Broca’s area, pars triangularis and primary somatosensory cortex (BA 45 and 3, channel #10; *d* = 1.42). In the category contrast, more areas had a higher activation in HC than in AD: left Broca’s area and DLPFC (BA 45 and 46), left Wernicke’s area (BA 40, channel #19, *d* = 1.29), left SMA (BA 6, channel #7; *d* = 1.20), and right DLPFC (BA 9 and 46).

The contrast between the HC and the bvFTD groups showed even more pronounced differences. In the letter contrast, wide areas of the frontoparietal cortex in both hemispheres showed stronger activation in HC than in bvFTD (DLPFC, SMA, Wernicke’s area, BA 9,6, 40, 39, channels left #18, 20 (*d* > 1.1), right 18, 20, 21, 22 (*d* > 1.3)). The more basal areas—such as the left MTG and right MTG, STG, and the temporopolar area—showed less activation in the HC group (left: BA 21, channel #1 (*d* = 1.43), right: BA 21, 22, 38, channel #3, 7, 2 (*d* > 1.1)). The overall pattern is similar in the category contrast, with more activation in left Wernicke’s area (BA 40, channel #14, 19; *d* > 1.1) as well as right DLPFC (BA 9 and 46, channel #14, 19; *d* > 1.1) and STG (BA 22, channel #13; *d* = 1.45) and less activation in left Broca’s area, SMA, and STG (BA 45, 6, 22, channels #3, 6, 7; *d* > 1.2) and in right MTG, STG, and Broca’s area (BA 21, 22, 45).

The contrast of the two demented groups (bvFTD vs. AD) was characterized by a nearly symmetrical pattern in both condition contrasts: Broca’s area was more activated in both condition contrasts in the bvFTD than in AD (BA 45, left: channel #8 in both conditions, #3 additional in the category condition; right: #6 in the category condition; *d* > 1.2), and right STG and temporopolar area in the letter contrast (BA 22, 38, channel #2, 7, *d* > 1.2). More activation in the AD group compared to bvFTD was found in the left primary somatosensory area (BA 3, channel #15, letter contrast; *d* = 1.24) and the neighboring left Wernicke’s area (BA 40, channel #14, category contrast; *d* = 1.44), as well as the right Wernicke’s area and the neighboring STG (BA 40, channel #17, additional in the letter contrast BA 39, channel #18, BA 22, channel #13 in the category contrast, respectively; *d* > 1.1) as well as in the superior parietal SMA (BA 6, channel #20, letter contrast; *d* = 1.23).

### Behavioral and fNIRS data

The analysis of the association between performance (produced words) and cortical activation (ROIs) showed significant correlations only for the AD group in the letter condition, with positive correlations indicating increased cortical activation with an increasing number of produced words. This was found for the left parietal ROI (*r* = 0.85, *p* = 0.007) and the right DLPFC (*r* = 0.84, *p* = 0.009); a negative correlation was found for the right and left Broca ROI (*r* = –0.84, *p* = 0.009, and *r* = –0.95, *p* < 0.001). Due to this surprising result, these four ROIs were then correlated with each other, and reached high significance levels corresponding to the positive or negative correlation, respectively, compared to behavioral data (see Table 3). In all other conditions and groups, no significant correlation could be found after Bonferroni-Holm correction.

**Table 3:** Correlations between the regions of interest in the Alzheimer’s dementia group for the letter condition

	Behavioral data		DLPFC left		Broca left		Parietal left		DLPFC right		Broca right	
	*r*	*p*	*r*	*p*	*r*	*p*	*r*	*p*	*r*	*p*	*r*	*p*
DLPFC left	0.66	0.075										
Broca left	–0.84	0.009	–0.84	0.009								
Parietal left	0.85	0.007	0.54	0.164	–0.88,	0.004						
DLPFC right	0.84	0.009	0.54	0.164	–0.89,	0.003	0.99	<0.001				
Broca right	–0.95	<0.001	–0.75	0.032	0.96	<0.001	–0.94	<0.001	–0.93	0.001		
Parietal right	0.63	0.095	0.98	<0.001	–0.80	0.018	0.51	0.195	0.50	0.200	–0.72	0.045

*DLPFC* dorsolateral prefrontal cortex

## Discussion

The results of this study indicate that cortical activation measured with fNIRS while performing a Verbal Fluency Task (VFT) differs between healthy elderly controls (HC) and subjects suffering from a neurodegenerative dementia such as Alzheimer’s dementia (AD) or the behavioral variant of frontotemporal dementia (bvFTD). Furthermore, this activation differs between the two types of neurodegenerative dementia, a result shown for the first time in task-related functional imaging and for the first time using fNIRS in bvFTD.

The difference between younger and older healthy controls in cortical activation during the VFT has been described previously [26, 30, 45]. Heinzel et al., who completed the largest fNIRS investigation to date in the healthy elderly, postulated that a reorganization of cognitive function via a compensatory mechanism taking over from the well-localized activation of the left DLPFC occurs with increasing age [26, 27]. This is in line with the findings of an increasing frontotemporal and parietal cortical activation in a smaller subgroup of the sample reported by Heinzel et al. [26]. Considering the functional aspect, the additional DLPFC activation suggests a further involvement of executive functions necessary for active maintenance of context or goal-related information during a task, as well as dual-tasking [44]. A similar additional recruitment of activated brain regions in the elderly, combined with a reduced asymmetry of activation in both hemispheres, has been described as the HAROLD effect (Hemispheric Asymmetry Reduction in OLDer adults) by Cabeza [46].

A reduction in cortical activation during VFT performance in AD patients compared to HCs has been shown previously. However, in this previous study, a smaller area was studied and a smaller local resolution was used, due to a probeset with only a few optodes [30, 47]. Nevertheless, the characteristics of the activation pattern were not substantially different from the HC, which we confirmed in a larger probeset in the current study.

As compared to the findings in the AD group, the current bvFTD group showed a remarkably different cortical activation pattern during VFT performance. Neither the compensating mechanisms, which have been found in healthy elderly subjects as mentioned above, with activation in frontotemporal areas, nor a weakened pattern, as in AD patients, could be identified for bvFTD patients. Broca’s area, instead of the DLPFC and the superior temporal gyrus, was activated in this group. Differential compensation mechanisms between the two neurodegenerative diseases could be a possible explanation for these different activation patterns. Whereas AD patients recruit a pattern of activated brain areas similar to healthy controls, bvFTD patients seem to activate a completely different pattern, perhaps suggesting more severe disorganization of the (frontal) cortex compared to AD, and perhaps also indicating a cause for the more altered behavior in bvFTD. The areas with the strongest structural impairments in bvFTD, the frontal and temporal cortices, seemed to be the most highly activated in this group. These activations may possibly have no functional effect, due to a more dysfunctional activation of impaired neurons. The results of a reduced glucose metabolism in these areas, measured by FDG-PET [48] as an indicator for reduced activity, contradict this hypothesis. Restrictively, FDG-PET measurements in bvFTD are normally not functional measurements, but instead map overall activity of brain areas. An explanation of this mismatch of FDG-PET and fNIRS measurements could be a reduced resting-state metabolism (measured by FDG-PET) in bvFTD patients combined with a high task-related activation indicating an increased cognitive effort.

A relevant dissimilarity between AD and bvFTD concerning cognitive function was also found in studies investigating resting state with fMRI. During resting state, the (relatively) posterior default mode network was more strongly impaired in AD and less so in FTD [13, 14]. By contrast, the more frontal salience network was more affected in FTD than in AD [15, 16]. Despite the difficulties of a comparison between resting-state and task-related brain activation, the striking frontal activation in bvFTD might be—following the postulated mechanisms in healthy elderly and AD—a correlate of a compensation mechanism.

At first glance, the positive and negative correlations between the ROIs and the behavioral data in the AD group seem to be contradictory. However, with positive brain-behavior correlations for the DLPFC and parietal cortex vs. negative correlations for Broca’s area, we further correlated activation between these different ROIs and consistently found negative correlations between ROIs of the frontoparietal cortex and Broca’s area (see Table 3). This indicates that, with increasing activation of structures within the frontoparietal control network [49], weaker activation occurred in language-specialized areas (i.e., Broca’s area) and vice versa. It therefore makes perfect sense that opposing correlations were observed between behavioral data and frontoparietal ROIs vs. Broca’s area. The results also indicate that stronger activation within frontoparietal control structures was associated with superior VFT performance, whereas weaker activation within these control areas—and concurrently increased activation in Broca’s area—was detrimental for the word production outcome in this task.

Nevertheless, there are limitations to this study. Due to this being a pilot study, we included only a small number of subjects. Furthermore, a heterogeneity in the groups of demented subjects arises as a result of the clinical diagnosis criteria. A relevant critical point is that the frontotemporal atrophy which is necessary for the diagnosis of bvFTD may lead to systematic artifacts in the measurement. This specific atrophy might hinder direct comparison between the single groups as atrophy patterns between AD and FTD differ substantially [35, 50]. Furthermore, a direct relation of functional and structural data is not possible as fNIRS is only able to record functional activity and not structural information. The possibility of a functional-structural link using MRI does not solve the problem of specific atrophy as the atrophy is quantifiable but the different atrophy pattern cannot be compensated to improve the comparability. It should also be mentioned that the AD and bvFTD groups were examined in the course of their diagnostic and/or therapeutic procedure in the hospital whereas the control group received their neuropsychological and fNIRS examination as part of a large assessment of a longitudinal study (TREND study). Due to the motivated participation, the behavioral data, and the healthy status of the TREND participants, a systematic impact of this difference on the fNIRS results appears unlikely.

Another limitation is the incomplete characterization of the FTD and AD groups by biomarkers. The subjects of the present study were diagnosed according to valid criteria in which biomarkers are not regularly included, so only a part of the FTD sample has a full biomarker set including C9orf72 or granulin genotypes, as both biomarkers show a significant influence on network connectivity as measured with fMRI [18, 19]. Lee et al. showed an increase of connectivity in the default-mode network in bvFTD patients without C9orf72 mutation vs. C9orf72 carriers but no differences in the salience network, which is characteristically more altered than the default-mode network in bvFTD patients [18]. Premi et al. compared Granulin mutation carriers (healthy and FTD patients) with non-carriers and found a reduced regional connectivity [19]. Hence, as the relevance of genetic variants on categorization of bvFTD is currently well known, the impact of genetic variants for functional imaging including fNIRS will be a focus over the next years.

A restriction for the comparison of cortical activation in demented vs. healthy subjects in general is the difference in behavioral results which are apparent in the contrast between subjects with and without dementia. Here, the behavioral data of the bvFTD subjects were used for matching AD subjects from a larger sample; therefore, the two demented groups are directly comparable. In spite of identical instruction, the imbalance of the produced words in the active and the control conditions in the demented groups is a source of artifacts (e.g., increased activation of Broca’s area in the control condition) and could not be explained in this study. Superficially considered, this problem does not exist in resting-state analysis; however, to what extent demented patients are able to follow the instruction for rest as the basis for resting-state measurements is even less measureable.

## Conclusion

Summarizing the results of this study, bvFTD can be successfully investigated with a task-related functional imaging design. Similar to resting-state studies which have pointed out differences in the established networks in bvFTD patients as compared to AD patients, clear differences concerning the activated areas were shown. While AD patients activated a “compensation” pattern similar to but weaker than the healthy elderly, mainly in the DLPFC but also in parietal areas, FTD patients did not show this known “compensation” pattern. More frontopolar-localized areas such as Broca’s area were active, possibly as an alternative compensation in a strictly different pathological process.

## Additional files

Additional file 1: Statistical source data for Fig. 2. Contrast of phonematic condition, semantic condition, and the control condition in the three groups; grey background signifies significant contrasts. *AD* Alzheimer’s dementia, *bvFTD* behavioral variant of frontotemporal dementia, *CAT* category (semantic) condition, *HC* healthy controls, *LET* letter (phonematic) condition, *WD* weekday (control) condition. (XLSX 13 kb)
Additional file 2: Statistical source data for Fig. 3. Contrast of two groups each concerning the contrast of phonematic condition, semantic condition, and the control condition; grey background signifies significant contrasts. *AD* Alzheimer’s dementia, *bvFTD* behavioral variant of frontotemporal dementia, *CAT* category (semantic) condition, *HC* healthy controls, *LET* letter (phonematic) condition, *WD* weekday (control) condition. (XLSX 13 kb)

## Abbreviations

AD: Alzheimer’s dementia
ANOVA: Analysis of variance
BA: Brodmann area
bvFTD: Behavioral variant of the frontotemporal dementia
CBSI: Correlation-based signal improvement
CERAD: Consortium to Establish a Registry for Alzheimer’s Disease
DLPFC: Dorsolateral prefrontal cortex
FDG-PET: 2-Deoxy-2-[18F]fluoro-d-glucose positron emission tomography
fMRI: Functional magnetic resonance imaging
fNIRS: Functional near-infrared spectroscopy
HC: Healthy controls
HHb: Deoxygenated hemoglobin
ITG: Inferior temporal gyrus
MTG: Middle temporal gyrus
O_2_Hb: Oxygenated haemoglobin
ROI: Region of interest
SMA: Supplementary motor area
STG: Superior temporal gyrus
TREND: Tuebinger evaluation of Risk factors for Early detection of NeuroDegeneration
VFT: Verbal Fluency Task
